# Nutritional Rehabilitation of HIV-Exposed Infants in Malawi: Results from the Drug Resources Enhancement Against AIDS and Malnutrition Program

**DOI:** 10.3390/ijerph9020421

**Published:** 2012-01-30

**Authors:** Ersilia Buonomo, Simona de Luca, Dyna Tembo, Paola Scarcella, Paola Germano, Anna Maria Doro Altan, Leonardo Palombi, Giuseppe Liotta, Karin Nielsen-Saines, Fulvio Erba, Maria Cristina Marazzi

**Affiliations:** 1 Department of Public Health, Tor Vergata University, via Montpellier, Rome 00133, Italy; Email: ersiliabuonomo@gmail.com (E.B.); paola.scarcella@alice.it (P.S.); annamariadoro@tiscali.it (A.M.D.A.); leonardo.palombi@gmail.com (L.P.); giuseppeliotta@hotmail.com (G.L.); erba@uniroma2.it (F.E.); 2 Department of Nutrition, Health Education Center, Perugia University, Perugia 06100, Italy; Email: simonadeluca.deluca@gmail.com; 3 Department of Nutrition, DREAM Program Malawi, Blantyre, Malawi; Email: tembodyna@yahoo.com; 4 DREAM Program, Community of Sant’ Egidio, Piazza S. Egidio 3a, Rome 00153, Italy; Email: dream@santegidio.org; 5 Department of Pediatrics, David Geffen School of Medicine at UCLA, MDCC 22-442, 10833 LeConte Ave, Los Angeles, CA 90095, USA; 6 Department of Preventive Medicine, LUMSA University (Libera Università Maria SS. Assunta), via della Traspontina 21, Rome 00193, Italy; Email: marazzi@lumsa.it

**Keywords:** HIV-exposed children, malaria, infant malnutrition, peer education, nutritional rehabilitation

## Abstract

Infant malnutrition in sub-Saharan Africa is a public health priority and a challenge in high HIV prevalence areas. The Drug Resources Enhancement Against AIDS and Malnutrition program, with multiple medical centers in Sub-Saharan Africa, developed an innovative intervention for the surveillance and control of malnutrition. In a pilot initiative, 36 HIV-exposed children were evaluated at baseline upon presentation for malnutrition and at six months post- treatment. Parameters included HIV-free survival, nutritional status and change in diet. Food diary data was entered and processed using the Nutrisurvey (WHO) software. At 6 months post-intervention, a significant improvement in anthropometric parameters was noted. Slowing of linear growth was observed in patients with malaria with a mean gain in centimetres of 4.4 ± 1.7 as compared to 5.6 ± 1.7 in children with no malaria, *p* < 0.048 (CL 95%: −2.32, −0.01). Dietary diversity scores increased from 5.3 ± 1.9 to 6.5 ± 1.3, *p* < 0.01 at 6 months. A significant increase (+25%, *p* < 0.02) in the number of children eating fish meals was noted. Our pilot data describes positive outcomes from a rehabilitative nutritional approach based on use of local foods, peer education, anthropometric and clinical monitoring in areas of high food insecurity. The relationship between malaria and linear growth retardation requires further investigation.

## 1. Introduction

The treatment of infant malnutrition in sub-Saharan Africa is a public health priority and represents a challenge in areas with high HIV/AIDS prevalence [1,2,3,4]. In Malawi, HIV and malnutrition are the two major causes of infant morbidity and mortality [5,6]. Malnourished children who do not quickly break away from the vicious cycle of infectious disease and growth failure are also vulnerable to irreversible cognitive damage [7]. Growth failure is partially attributable to inadequate complementary feeding [8,9]. Educational interventions have proven to be effective in changing the behavior of caregivers with respect to the care of malnourished children [10,11]. There is a strong association between maternal education and reduction of infant mortality with positive public health outcomes [12]. Therefore, the implementation of nutritional counselling programmes, through which mothers can be given appropriate tools to enable proper nutrition and rehabilitation of malnourished children, is crucial [13]. Children born to HIV-positive women are particularly susceptible to malnutrition. Growth failure and wasting are frequently found in this population, especially in areas where high prevalence of HIV/AIDS co-exists with high rates of food insecurity [14,15]. 

Interventions aimed at preventing mother-to-child HIV transmission in the absence of maternal highly active antiretroviral therapy during breastfeeding have produced disputable results in terms of the control of infant mortality and malnutrition, particularly in regards to infant HIV-free survival [16,17,18]. Maternal antiretroviral treatment as a prophylactic measure for prevention of perinatal HIV transmission during pregnancy and while breastfeeding represents a breakthrough in prevention science, curtailing the spread of the epidemic in children and containing infant mortality and malnutrition [19,20]. Following review of recent HIV perinatal guidelines, the WHO proposed an amendment to their current protocol which provides for the administration of antiretroviral therapy to mothers during lactation period [21]. Child malnutrition nevertheless remains a challenge to the health of HIV-exposed infants requiring the implementation of sustainable and locally-acceptable interventions in areas with limited resources [22]. The Drug Resource Enhancement Against AIDS and Malnutrition Program, also known as DREAM, sponsored by the Sant’Egidio Community, is pioneer in the prevention of mother-to-child transmission of HIV/AIDS in Sub-Saharan Africa, with 80,000 HIV-infected and/ or exposed individuals in care. At DREAM medical centers, the HIV perinatal protocol integrates, at no charge, antiretroviral therapy with health education and nutritional supplementation during pregnancy and lactation [23,24]. This has resulted in significant infant HIV-free survival rates exceeding 90% at 18 months of age [24]. Infants born in the program are monitored until 18 months of age through monthly checks. In the event of signs of malnutrition, affected children are placed in a tailored malnutrition-control program. The approach entails intensive rehabilitative treatment, for improvement of infant nutritional status, as well as an educational program for the mother or caregiver, so that the child’s diet can be improved. 

In the present study we describe the nutritional rehabilitation of 36 HIV-exposed infants followed at a DREAM centre in Malawi, who were evaluated at the onset of malnutrition and again after six months for HIV-free survival, growth, nutritional status and change of diet, following implementation of our intervention. 

## 2. Sample and Methods

### 2.1. Procedures

During the month of March 2009, all HIV-exposed children followed at the Blantyre DREAM center in Malawi, who presented with severe or moderate acute malnutrition, were offered participation. This totalled 36 HIV-exposed children within a cohort of 671 children (5.4%) followed at our center during that time. The 36 infants with malnutrition included 26 males, with a mean age of 12 months ± 3.04. Infants were enrolled following parental informed consent. The cohort was examined at the time of onset of malnutrition (T0) and following six months of treatment (T1) to assess the impact of the intervention on infant malnutrition and health. At baseline, all children were HIV-uninfected based on virus load testing and none of them were being breastfed, though all had received exclusive breast milk until six months of age. All mothers were receiving monthly nutritional supplementation through the program and all came from low socio-economic households as shown in Table 1.

**Table 1:** Demographic, social and housing features of the study population.

Demographic, social and housing features of the study population	%
Inadequate housing	27%
Access to drinking water	22%
Access to hygienic toilets	6%
Access to electrical power	28%
Maternal illiteracy	11%
Unemployment	84%

The study setting was the Blantyre Center, operated by the DREAM program, a public health program aimed at prevention and treatment of HIV/AIDS with multiple centers in 10 African countries. This specific study was approved by the local institutional review board, the Malawi National Health Sciences Committee (NHSC). The DREAM program’s approach to the diagnosis, treatment and prevention of HIV/AIDS in Africa has been documented in prior studies [25,26,27]. Because HIV-infected women breastfeed until 6 months of age while receiving antiretroviral therapy as part of program guidelines, large cohorts of infants have demonstrated increased survival, lower levels of postnatal HIV acquisition and lower prevalence of growth failure at 6, 12 and 18 months similar to or lower than the national average for non-HIV-exposed infants [27,28,29]. 

### 2.2. DREAM Malnutrition Program

Within the focus of prevention of HIV-mother-to-child transmission, surveillance, monitoring and treatment of malnutrition in HIV-exposed infants is performed by the program. Anthropometric and clinical checks of HIV-exposed children are performed monthly and measures are registered through a software package obtained in collaboration with the Department of Nutrition of the World Health Organization, which incorporates certain features of the WHO Anthro software [30,31]. Nutritional support to participants is provided throughout the duration of pregnancy and postpartum and to the infant from weaning to 18 months of age. Nutritional support during pregnancy aims to reduce rates of premature delivery and infant low birth weight, while encouraging adherence to antiretroviral regimens. Monthly nutrition packs contain corn flour, rice, beans, peanuts, vegetable oil, sugar and during the weaning period rice flour. For children presenting with varying degrees of moderate to severe malnutrition, levels of supplementation also vary, with the addition of *likuni phala*, a locally-produced corn and soya flour blend. In severe acute cases, therapeutic foods are also used, in accordance with Malawian guidelines for treatment of severe malnutrition [32]. Vitamins and micronutrient supplementation are provided to all children with moderate to severe malnutrition, based on national guidelines as well as clinical assessments. 

### 2.3. Nutritional Counselling

Mothers of infants admitted to nutritional rehabilitation are placed in an educational program targeting change in eating habits and dietary diversity with a focus on the nutritional status of children and increase in energy density. A multidisciplinary team consisting of a counsellor, nurse, dietician and two HIV positive women working as DREAM activists provide nutritional education mothers. Counselling and education sessions cover topics as below:

- Vicious cycle of HIV, malnutrition, infection;
- Macronutrients, vitamins, micronutrients: their functions and needs;
- Food groups and dietary diversity;
- Health and a balanced diet;
- Feeding of healthy and sick children;
- Nutritional care of malnourished children;
- Promotion of breastfeeding;
- Nutritional advice on weaning and complementary feeding;
- Water and food hygiene.

### 2.4. Anthropometric Assessments and Classification of Malnutrition

In accordance with international standards, anthropometric parameters assessed include weight, height, arm circumference and the presence of bilateral pre-tibial edema. Children are measured without clothing or footwear; weight (in kilograms) is determined using a mechanical baby scale (SECA 745—Class III), while height (in centimeters) is assessed by laying children on their back and using a stadiometer for newborn babies and infants (SECA 210). Staff involved in assessing measurements is appropriately trained. Weight and height data is routinely inputted into the DREAM program software, updated on the basis of WHO’s 2006 child growth standards featuring graphing and anthropometric utilities incorporated from the WHO/Anthro package [33]. The software is able to generate graphs of respective growth curves and indicates cut-off points in different colors. Children with weight for height z-scores (WHZ) < −2 and/or middle upper-arm circumference (MUAC) between 115 mm to 119 mm and/or weight for age z-score (WAZ) < −3 z-score and/or two consecutive static or declining weights are identified as children with Moderate Acute Malnutrition (MAM), while Severe Acute Malnutrition (SAM) is characterized by a WHZ < −3 and/or MUAC <115 mm and/or presence of bilateral edema, in accordance with WHO/UNICEF criteria and the Malawian guidelines for the management of malnutrition [32,33,34,35]. Assessment of edema and clinical conditions is performed by health center clinicians (doctors and clinical officers). Findings are entered into the computerised medical records system. Features of the DREAM program software package have been previously described [30]. 

### 2.5. Food Survey and Calculation of Nutritional Intake

In order to assess eating habits of study subjects, trained staff utilized a 24-hour food recall diary, a food diversity index (the Dietary Diversity Score or DDS) and a food atlas. This allowed a qualitative and semi-quantitative assessment of the infants’ diet. The purpose of the food atlas was to conduct a quantitative dietary assessment (an estimate of the quantity of foods consumed). The atlas was prepared especially for the program describing local foods for the study population in local dialects. Caregivers selected pictures of the foods most frequently eaten, and the atlas demonstrated different portions, enabling estimates of food consumption through the use of pictures of local measuring utensils (*chipande*). Previously weighed portions of foods were shown to mothers who selected out the food quantity that matched the amounts they were providing. This procedure enabled guardians to recall the quantity of food consumed and assisted staff in the calculations of dietary intake and subsequent nutritional counselling support. Dietary diversity was assessed using the Dietary Diversity Score (DDS). The index evaluates dietary diversity with reference to the 12 food groups used by FAO for developing countries. Several studies demonstrate a positive association between dietary diversity and nutritional status of children [36,37]. Food diary data for the infants was entered and processed using the Nutrisurvey (WHO) software [38], a package for Windows XP/Vista/2007 previously used in similar studies [38,39]. A value of less than 75% of the FAO Recommended Nutritional Intake was considered inadequate [40,41]. Nutrisurvey enabled the addition of other foods to the database, therefore local Malawian foods were included based on their nutritional composition as determined from international or Malawian sources [42,43].

### 2.6. Statistical Analysis

Data was processed using the Statistical Package for Social Sciences software (SPSS for Windows, version 19.0, 2010, Chicago, Illinois) [44]. Analysis of all anthropometric and nutritional variables was performed at two time intervals, on admission and at six months. Comparison of anthropometric variables was performed through paired-sample t-test with a correlation analysis. The degree to which high incidence of malaria infection influenced changes in infant growth was analyzed using an independent sample t-test with a 95% confidence interval for the difference. Nutritional intake and dietary diversity were calculated by means of a paired-sample test. 

## 3. Results

Thirty-six HIV-exposed children were diagnosed with acute moderate to severe malnutrition during the month of March 2009. Nutritional characteristics of the population on enrollment and at follow-up are illustrated in Table 2. 

**Table 2:** Anthropometric characteristics of study subjects at baseline and on follow-up.

Anthropometric characteristics of study subjects at baseline and on follow-up.	Admission (n = 36)	6 month follow-up (n = 36)	*p*-value *
Age (months), mean (SD)	11.8 ± 3.0	17.7 ± 2.9	
Weight, mean (SD) kg	6.7 ± 0.91	8.2 ± 0.92	<0.0001
Height, mean (SD) cm	67.4 ± 3.87	72.4 ± 3.44	<0.0001
Middle upper arm circumference, mean (SD) cm	12.8 ± 1.21	14.58 ± 1.25	<0.0001
Weight for age z-score, mean (SD)	−2.96 ± 0.77	−2.31 ± 0.73	<0.0001
Height for age z-score, mean (SD)	−3.03 ± 1.13	−3.31 ± 0.96	0.011
Weight for height z-score, mean (SD)	−1.78 ± 1.06	−0.88 ± 0.83	0.007
Children with bilateral edema n. %	4 (11)	0	
Children with Severe Acute Malnutrition, n (%) (MUAC <115 mm and/or Bilateral oedema and/or WHZ < −3)	12 (33)	0	
Children with Moderate Acute Malnutrition, n (%) (MUAC < 119 mm and/or WHZ < −2 and/or WAZ < −3 and/or two consecutive static or declining weights)	24 (67)	6 (16)	
Growth Velocity z-score at follow-up, mean (SD)		−1.27 ± 1.3	

* T-test for paired samples.

On admission, 12 children (33%) were severely malnourished and 24 (67%) were moderately malnourished, according to local and WHO/UNICEF criteria. The nutritional status six months later was overall improved, with none of 12 children originally in a state of severe acute malnutrition having persistent findings, although 6 subjects (16%) with moderate acute malnutrition still fulfilled criteria for this condition. All children remained HIV-uninfected at the time of discharge and there was no mortality. At the six month follow-up visit, there was a statistically significant gain in body weight (kg), height (cm), MUAC (mm), weight for age z-scores and weight for height z-scores. Linear growth velocity slowed, as also corroborated by the statistically significant decline in height-for-age z-score values. The growth delay observed was partly attributable to malaria infection with a significant difference in actual centimeters gained by children with malaria as compared to those who had had no episodes of malaria, as shown in Table 3. 

**Table 3:** Growth velocity z-score according to malaria status.

Growth velocity z-score according to malaria status	Children with malaria (n = 17)	Children without malaria (n = 19)	*P*-value *
Height gain at follow-up, mean (SD)	4.4 ± 1.7	5.6 ± 1.7	*p* < 0.048 (CL95% 2.32 − 0.01)
Growth Velocity z-score at follow-up, mean (SD)	−1.6 ± 1.2	0.82 ± 1.2	*p* < 0.046 (CL95% 1.7 − 0.01)

* T-test for independent samples.

Children who experienced no episodes of malaria were significantly taller than those who had the infection, as shown in Table 3. In addition, the growth velocity was significantly lower in children with episodes of malaria. 

Nutritional characteristics of the population at entry and follow-up in regards to number of meals, dietary diversity score and intake of macronutrients are illustrated in Table 4. 

**Table 4:** Dietary characteristics of the population.

Dietary characteristics of the population	Admission	Follow-up	*p*-value
Number meals, mean (SD)	4 ± 1	5 ± 1	0.050
Dietary diversity score, mean (SD)	5.3 ± 1.9	6.5 ± 1.3	0.012
Energy, mean (SD), Kcal	916 ± 391	1,180 ± 342	0.003
Proteins, mean (SD), g	31 ± 17	42 ± 16	0.010
Fats, mean (SD), g	27 ± 19	35 ± 18	NS
Carbohydrates, mean (SD), g	135 ± 55	170 ± 51	0.006
Percentage of children with energy intake lower than 75% of Required Daily Amount-RDA (WHO/FAO) n (%)	14 (39)	10 (27.8)	
Percentage of children with protein intake lower than 75% of Required Daily Amount-RDA (WHO/FAO) n (%)	4 (11)	0	

The food survey, conducted prior to nutritional counselling, revealed that children were being fed only a limited number of food groups; the dietary diversity value on admission was 5.3 ± 1.9, and at follow-up, 6.5 ± 1.3, representing a statistically significant difference. It is interesting to note the significant increase in the mean number of meals, which rose from 4 to 5 meals per day on average. For comparative analysis of the dietary composition in terms of energy and nutrients, a rise in the intake of energy and all macronutrients was observable, with a statistically significant increase in caloric, protein and carbohydrate intake. At follow-up, 28% of children were still receiving less than 75% of the daily allowance of recommended calories per guidelines [40], while all children received at least 75% of the protein allowance at that time point [41]. 

**Figure 1:**
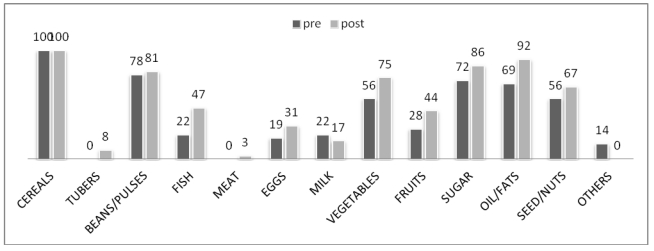
Percentage of children receiving foods at baseline and at follow-up based on 24-h dietary recalls.

One of the major problems evident at baseline was that subjects had a monotonous diet. Analysis of food recalls before and after the intervention showed significant changes in terms of dietary diversity, as indicated in Figure 1. The number of children consuming foods of animal origin such as fish, eggs and meat increased by 25% (*p* < 0.02), 12% and 3% respectively. The consumption of foods of plant origin increased 19% for vegetables, 16% for fruit and 11% for nuts and seeds. The dietary assessment revealed a statistically significant improvement in the consumption of fish and oil/fats, probably as a result of nutritional education. This was particularly evident for fish consumption, as the DREAM program does not include this food type in its nutrition packs. The increased consumption of fish was also consistent with the local diet, as Malawi is a country rich in freshwater fish, such as sun dried *E. Sardella* and *C. Inornatus*, high-energy and high-protein fish products [43]. During the educational sessions, mothers or guardians were strongly encouraged to enrich the various porridges that are typical of the Malawian infant diet with fish meals, made from small, dried or ground fish. Another noticeable effect of nutritional education was the increased consumption of fruit and vegetables, foods often spurned as non-nutritious in Malawi. 

## 4. Discussion

Our primary objective in this pilot project was to assess the impact of a health-driven, growth monitoring intervention coupled to health education and food supplementation on the diet and nutritional status of malnourished HIV-exposed infants. Our findings demonstrate that both nutritional status and diet improved significantly over the course of a short observation period. The most significant improvement was noted in cases of acute malnutrition. Our results are consistent with those of other studies and highlight the complexity of nutritional rehabilitation efforts in the early years of life particularly in areas where chronic and moderate deficits abound, confounded by high rates of food insecurity and infectious diseases such as HIV and malaria [45].

Food insecurity is an ongoing problem in Malawi, especially in rural areas, where mothers work in the fields most of the day, resulting in no time availability for the preparation of adequate meals for their children. Despite these inherent difficulties, once the educational sessions were completed, mothers demonstrated a greater attention to their children’s diet and had increased awareness of malnutrition and its consequences. It was clear that mothers understood, following completion of the intervention, that in the absence of infections, such as HIV, the issue of nutrition is solely related to dietary habits. One strong motivator for participants was that their children were not HIV infected like themselves because of their enrolment into our program during pregnancy. As such, they wanted ensure that they would not succumb to malnutrition having avoided HIV infection. Raising maternal awareness rendered women actively involved and enthusiastic during the educational sessions, a factor likely associated with improvement in the nutritional status of the entire cohort.

A key factor for program effectiveness is the approach to combat malnutrition based on local foods [46]. Within our centers, nutritional supplementation is based on local foodstuffs [47]. Therapeutic foods were only used in our subjects in cases of severe acute malnutrition and for a period not exceeding 30 days. Nutritional counselling to mothers took the form of peer education by local staff in the local language based on a diet consisting of local foods. In addition nutritional education sessions were performed weekly and also included practical demonstrations of cooking with local ingredients. The extended educational process, of at least six intensive months followed by 12 non-intensive months, enabled mothers to change their eating habits themselves, understand their mistakes and adhere to advice given by our counsellors and activists during visits to the center and home visits.

The statistical analysis of anthropometric data confirms the complex clinical and nutritional profile of our pediatric subjects and highlights positive outcomes in terms of HIV-free survival and reduction in malnutrition. HIV-exposed children often exhibit particular susceptibility towards malnutrition and infection, resulting both from a compromised maternal immunologic condition and their social vulnerability. Numerous studies have reported high rates of infant mortality associated with malnutrition due to problems resulting from weaning triggered by poverty in the affected families [48,49]. 

Our population did not reach high levels of malnutrition because all infants and mothers received nutritional supplementation and also because the monitoring system enabled early detection of children with low anthropometric indices triggering an intense intervention over an extended period of time. As a consequence anthropometric parameters (weight and height) improved. Our intervention, however, did not succeed in impacting chronic malnutrition, as infants in fact demonstrated signs of significant stunting. In our opinion, however, stunting in our patients did not appear to be associated with nutritional challenges but with a high incidence of malaria episodes. Malaria represents a serious risk to the life and health of children in sub-Saharan Africa and has, for a long time, also been identified as a factor linked to stunting. Stunting appears to be associated primarily with the high levels of anorexia and lethargy brought on by malaria attacks. For years, numerous studies conducted in areas of endemic malaria have found a significant association between malaria episodes and low height-for-age z-scores in children [50,51]. In our study, the positive association between stunting and malaria corroborates previous reports illustrating the serious impact of infectious diseases on the growth and development of children in areas with limited resources [52,53].

## 5. Conclusions

Our results point to the effectiveness of nutritional rehabilitation interventions when integrated within an HIV mother-to-child transmission prevention program. Interventions based on supplementation with local foods, peer-to-peer nutritional education, and software-assisted clinical and nutritional monitoring are key factors. As a consequence, children in our study achieved good levels of dietary diversity and a satisfactory quantitative/qualitative diet which was significantly associated with improvement of anthropometric parameters. Malaria episodes were identified as a significant risk factor for reduced linear growth and, consequently persistence or onset of chronic malnutrition. Although our study is limited by a small sample size and the absence of a control group, the prospective, detailed evaluation described herein mirrors the large scale rehabilitative approach implemented by the DREAM program in our centers in Sub-Saharan Africa. Our pilot data demonstrates several positive repercussions on the health and nutritional status of HIV exposed infants in area with high rates of food insecurity. 
